# Network extreme eigenvalue: From mutimodal to scale-free networks

**DOI:** 10.1063/1.3697990

**Published:** 2012-03-29

**Authors:** N. N. Chung, L. Y. Chew, C. H. Lai

**Affiliations:** 1 Temasek Laboratories, National University of Singapore, Singapore 117508; 2 Division of Physics & Applied Physics, School of Physical & Mathematical Sciences, Nanyang Technological University, 21 Nanyang Link, Singapore 637371; 3 Beijing-Hong Kong-Singapore Joint Centre for Nonlinear and Complex Systems (Singapore), National University of Singapore, Kent Ridge 119260, Singapore; 4 Department of Physics, National University of Singapore, Singapore 117542

## Abstract

The extreme eigenvalues of adjacency matrices are important indicators on the influence of topological structures to the collective dynamical behavior of complex networks. Recent findings on the ensemble averageability of the extreme eigenvalue have further authenticated its applicability to the study of network dynamics. However, the ensemble average of extreme eigenvalue has only been solved analytically up to the second order correction. Here, we determine the ensemble average of the extreme eigenvalue and characterize its deviation across the ensemble through the discrete form of random scale-free network. Remarkably, the analytical approximation derived from the discrete form shows significant improvement over previous results, which implies a more accurate prediction of the epidemic threshold. In addition, we show that bimodal networks, which are more robust against both random and targeted removal of nodes, are more vulnerable to the spreading of diseases.

Network extreme eigenvalues are succinct descriptors of the influence of the underlying topological structure of a complex network on its dynamics. This makes them important predictors of epidemic threshold of infectious diseases that propagate within real world complex network. Indeed, the recent demonstration that these eigenvalues are ensemble averageable has provided further support for this view. In this paper, we study into the ensemble averageability of extreme eigenvalues through a new perspective: the connection between multimodal network and scale-free network. The discrete nature of the multimodal network has allowed us to arrive at an improved analytical expression of the extreme eigenvalue for scale-free networks. The extreme eigenvalues calculated from our analytical expression are found to closely correspond to those obtained numerically, thus making significant improvement over earlier versions. The implication is a more accurate estimate of epidemic threshold, which is important for the elucidation of how vulnerable a particular network structure is to epidemic spreading. Our results are also applicable to the evaluation of strategies that aim to contain the spread of infectious diseases through the adjustment of the topology of network structures.

Many concepts in network science are well recognized as fundamental tools for the exploration on the dynamics of complex systems. In particular, scale-free networks have been widely used to describe and model diverse social, biological, and economic systems.^1–5^ In an ensemble of scale-free networks, it is known that the degree distribution of the nodes remains invariant. However, the topological structure can be distinct with different connection arrangements between the nodes in each network configuration. Such structural diversity can lead to discrepancies in the dynamics of individual network. Since the structural influences on certain dynamical processes are governed by the extreme eigenvalues of the network adjacency matrices,^6–13^ deviations in the extreme eigenvalues in network ensembles are of increasing interest in deciphering the underlying structural changes. Recently, it was found that the extreme eigenvalues of adjacency matrices, despite fluctuating wildly in an ensemble of scale-free networks, are well characterized by the ensemble average after being normalized by functions of the maximum degrees.^14^ Specifically, it had been proven that the probability of having a large variation in extreme eigenvalues in the ensemble diminishes as the size of the network increases. Considering the rich assortment of possible structural configurations of scale-free networks in an ensemble, this averageability is significant as it implies that dynamical processes which are governed by the extreme eigenvalues can be simply described by means of the ensemble average without the need to incorporate the connection details of the individual network. In particular, the average ability of a network to synchronize and its mean epidemic spreading threshold are shown to be well approximated by functions of the ensemble average of the eigenvalues. Therefore, finding a way to determine the ensemble average of the extreme eigenvalues becomes crucial in uncovering the topological influences of the network structure on a number of network dynamical processes.

In this paper, we investigate into the extreme eigenvalue of undirected scale-free network through its discrete form: the multimodal network. Note that for directed network, the extreme eigenvalue can be obtained from Refs. 15 and 16. Based on mathematical properties of the multimodal network, which are more tractable, we determine analytically the ensemble average of the extreme eigenvalues and investigate into circumstances under which the individual network can be better represented by its ensemble average. Interestingly, our results have enabled us to explore into the difference between bimodal^17,18^ and scale-free networks in terms of the ensemble average of the extreme eigenvalue.

Let us begin with a brief introduction of multimodal networks. In Ref. 19, a multimodal network with *m* modes is shown to contain *m* distinct peaks, which is exemplified by the degree distribution: $P(k)=\sum_{i=1}^{m}r_{i}\delta (k-k_{i})$. Note that δ(x) is the Dirac’s delta function. The discrete degrees of the network are $k_{i}=k_{1}b^{-(i-1)}$ with i=1,2,…,m. In addition, the fraction of nodes of degree $k_{i}$ is $r_{i}=r_{1}a^{-(i-1)}$. It is assumed that a>1 and 0<b<1 such that the degree distribution of the multimodal network follows a power law: $P(k_{i})=r_{i}\propto k_{i}^{-\beta }$. Hence, $r_{1}>r_{2}>\ldots >r_{m}$ for $k_{1}<k_{2}<\ldots <k_{m}$. As m→∞, the multimodal network converges to a scale-free network. The largest degree of the network is $k_{m}$, the smallest degree is $k_{1}$ which is between 1 and ⟨k⟩, and we have $b={\left(k_{1}/k_{m}\right)}^{\frac{1}{m-1}}$. Finally, the rest of the parameters are determined through the following equations:

$$\sum_{i=1}^{m}r_{i}=r_{1}\sum_{i=1}^{m}a^{-(i-1)}=1,$$ (1)

$$\sum_{i=1}^{m}k_{i}r_{i}=k_{1}r_{1}\sum_{i=1}^{m}{\left(\mathrm{ab}\right)}^{-(i-1)}=\langle k\rangle .$$ (2)

We follow the method outlined in Ref. 20 to find the maximum eigenvalue $\lambda_{H}$ of the network after we have determined all the parameters of the multimodal network. Let *G* be a graph with vertices described by the set $V(G)=\{v_{1},v_{2},\ldots ,v_{m}\}$; and let *A* represents the adjacency matrix. For each positive integer *n*, the number of different $v_{j}\to v_{i}$ walk of length *n*, denoted by $y_{j\to i}(n)$, is the ( *j*, *i*)th-entry in the matrix $A^{n}$. Note that two u→v walks: $W=(u=u_{0},u_{1},\ldots ,u_{k}=v)$ and $W'=(u=v_{0},v_{1},\ldots ,v_{l}=v)$, in a graph are equal if *k* = *l* and $u_{i}=v_{i}$ for all *i*, with 0≤i≤k.^21^ For example, $W_{1}=(v_{0},v_{1},v_{0},v_{2})$ is different from $W_{2}=(v_{0},v_{2},v_{0},v_{2})$ and both should be included in the calculation of $y_{0\to 2}(3)$. In other words, $y_{j\to i}(n)$ is the total number of all possible walks of length *n* from node *j* to *i*, including those that go backwards. Take a fully connected network with 3 nodes as an example, there are 3 walks of length 3 from node 1 to node 2, i.e., 1→2→1→2,1→3→1→2 and 1→2→3→2. This corresponds to ${\left(A^{3}\right)}_{12}=3$. In the eigen-decomposed form, we have $A^{n}=vD^{n}v'$, where *v* is a square matrix whose columns are the eigenvectors of *A,* and v' denotes the inverse of *v*. *D* is the diagonal matrix whose diagonal elements are the corresponding eigenvalues, i.e., $D_{\mathrm{ll}}=\lambda_{l}$. Hence,

$$y_{j\to i}(n)={\left(A^{n}\right)}_{\mathrm{ji}}=\sum_{l}\lambda_{l}^{n}v_{j,l}{v'}_{l,i}.$$ (3)

Note that Eq. (3) gives a summation over the *n*th power of the eigenvalues. When *n* is sufficiently large, the *n*th power of $\lambda_{H}$ dominates over the *n*th power of each of the remaining eigenvalues. Therefore, $y_{j\to i}(n)$ can be simply approximated by $\lambda_{H}$ as follow:

$$y_{j\to i}(n)\approx \lambda_{H}^{n}v_{j,H}{v'}_{H,i}.$$ (4)

Now, if we were to consider the number of walks of length *n* + 2 which start and end at node *H,*

$$y_{H\to H}(n+2)=y_{H\to H}(n)y_{H\to H}(2)+\sum_{j\neq H}y_{H\to j}(n)y_{j\to H}(2),$$ (5)

then according to Eq. (4),

$$\lambda_{H}^{2}\approx y_{H\to H}(2)+\sum_{j\neq H}\frac{y_{H\to j}(n)y_{j\to H}(2)}{y_{H\to H}(n)}.$$ (6)

The first term on the right hand side of Eq. (6) corresponds to the number of nearest neighbors of node *H*, i.e., the largest degree of the network, $k_{H}$. In Ref. 20, the second term on the right hand side of Eq. (6) is shown to be very small numerically for scale-free networks and is hence neglected. Since we are interested in finding a better approximation to the ensemble average of the maximum eigenvalues, we retain the second term and evaluate it by means of a statistical approach.

For this, we consider the walks which start from node *H*. Beginning from node *H*, the total number of all possible walks of length *n* to any node in the network is

$$y_{H}(n)=\sum_{j}y_{H\to j}(n)=\sum_{j}{\left(A^{n}\right)}_{\mathrm{Hj}}.$$ (7)

Since out of a total of N⟨k⟩ in-links and out-links (with *N* being the total number of nodes in the network), $k_{j}$ of them are directed into the node *j*, the fraction of walks that terminates at node *j* can be approximated by $\frac{k_{j}}{N\langle k\rangle }$. Therefore,

$$y_{H\to j}(n)\approx y_{H}(n)\frac{k_{j}}{N\langle k\rangle }$$ (8)

and

$$y_{H\to H}(n)\approx y_{H}(n)\frac{k_{H}}{N\langle k\rangle }.$$ (9)

From node *j*, the number of possible one-step walk is equal to the number of neighbors of node *j*, i.e., $y_{j}(1)=k_{j}$. Similarly, from a neighbor of node *j*, say $j_{1}$, the number of one-step walk is equal to the number of neighbors of node $j_{1}$, i.e., $y_{j_{1}}(1)=k_{j_{1}}$. Walking two steps from node *j* is the same as walking one step from the neighbor of node *j*. Hence,

$$y_{j}(2)=\sum_{q=1}^{k_{j}}y_{j_{q}}(1)\;=\sum_{q=1}^{k_{j}}k_{j_{q}}\;=k_{j}k_{j}^{\left(1\right)},$$ (10)

where $k_{j}^{\left(1\right)}=\sum_{q=1}^{k_{j}}k_{j_{q}}/k_{j}$ is the average degree of the first nearest neighbors of node *j*. The fact that node *j* is one of the neighbors of node $j_{q}$ implies that among the two-step walks that begin from node *j*, all walks that go from node *j* through its neighbors, and then go back to node *j*, are included. This means that backward walks are included in the calculation of $y_{j}(2)$ in Eq. (10). Therefore,

$$y_{j\to H}(2)\approx k_{j}k_{j}^{\left(1\right)}\frac{k_{H}}{N\langle k\rangle }.$$ (11)

Since $k_{j}k_{j}^{\left(1\right)}$ can be small, the approximation in Eq. (11) may not be precise for each *j*. Hence, the approximation is applicable only as an average over all nodes with degree $k_{j}$ instead of being valid for each individual case.

Next, we substitute Eqs. (8), (9), and (11)into Eq. (6). Then, we consider the multimodal property of the network. For multimodal scale-free network, there is a finite number *m*, of distinct degrees $k_{i}$, each with probability $r_{i}$. Thus,

$$\lambda_{H}^{2}\approx k_{m}+\sum_{i=1}^{m}R_{i}k_{i}^{2}\frac{k_{i}^{\left(1\right)}}{\left\langle k\right\rangle },$$ (12)

where

$$R_{i}=\left\{ \begin{array}{cc} r_{i} & \mathrm{for}1\leq i\leq m-1,\\ r_{i}-1/N & \mathrm{for}i=m. \end{array}\right.$$ (13)

Equation (12) implies that $\lambda_{H}$ depends on the specific way the nodes within the network are connected, which can differ broadly across the ensemble. When the exponent β of a scale-free network is small, the degree distribution is more heavy-tailed. The result is a larger variation in the distribution of $k_{i}^{\left(1\right)}$ in the network ensemble. Hence, there is greater deviation in the values of $\lambda_{H}$ in the ensemble. For multimodal network with fixed $k_{1}$ and $k_{m}$, the parameter *b,* and hence $k_{i}$, is fixed. In order to have a larger value of ⟨k⟩, the fraction of large-degree node has to be higher and the fraction of small-degree node has to be lower. This results in a more heavy-tailed distribution with a smaller value of β. In other words, β∝1/⟨k⟩. When the network size is larger, ⟨k⟩ has to be smaller for a fixed value of $k_{m}$, which arises from the general result $k_{m}\propto \sqrt{\langle k\rangle N}$.^2,22–24^ Hence, β is larger and the probability of having larger-degree node drops rapidly. This means that the values of $\lambda_{H}$ in an ensemble of multimodal networks deviate less as the networks become more sparse. In an ensemble of sparse networks, the individual network can thus be well represented by the ensemble average.

On the other hand, for a fixed value of ⟨k⟩, the degree distribution of multimodal networks varies with different values of $k_{1}$. Specifically, $\beta \propto k_{1}$. For two multimodal networks *A* and *B* of the same size, but having different values of $k_{1}$, the fraction of large-degree node for the network with smaller $k_{1}$, say network *A*, has to be larger in order for it to have the same average degree as network *B*. Thus, $\lambda_{H}$ of network *A* is larger. In other words, the choice of different values of $k_{1}$ can lead to different values of $\lambda_{H}$. Specifically, $k_{1}=1$ gives the extreme eigenvalue that is the largest, and $\lambda_{H}$ decreases as $k_{1}$ increases (see Fig. 1). In addition, since the degree distribution is more heavy-tailed for ensembles with smaller $k_{1}$, deviation in the extreme eigenvalues is larger. Note that previous results in Ref. 14 had shown the ensemble averageability of network eigenvalues for networks with $k_{1}\geq 3$. However, it had been shown in Refs. 25 and 26 that a smaller minimum degree can give rise to a broader distribution of the extreme eigenvalues of a network ensemble. Typically, the distribution of extreme eigenvalues of certain real-world networks has been observed to exhibit multimodal characteristics. This is consistent with our result that variation in the extreme eigenvalues is larger for an ensemble with a smaller $k_{1}$. Therefore, for network ensembles with $k_{1}<3$, the ensemble averages of the extreme eigenvalues have to be used with care.

After the qualitative discussion on the dependence of deviation in extreme eigenvalue on the deviation in the distribution of $k_{i}^{\left(1\right)}$, we next proceed to approximate the average values of $k_{i}^{\left(1\right)}$ analytically. For a random network, the average of the sum of the nearest neighbor degree is z2=G0'(1)G1'(1).^27^ Note that $G_{0}(x)=\sum_{k=0}^{\infty }p_{k}x^{k}$ is the generating function for the probability distribution of the node degree, while $G_{1}(x)=\sum_{k=0}^{\infty }kp_{k}x^{k}/\langle k\rangle$ is the generating function for the distribution of the degree of the vertices which we arrive at by following a randomly chosen edge. Hence, for a random multimodal network without any degree-degree correlation, $k_{i}^{\left(1\right)}\approx \langle k^{2}\rangle /\langle k\rangle$ and

$$\begin{array}{c} \langle \lambda_{H}\rangle =\sqrt{k_{m}+\sum_{i=1}^{m}R_{i}k_{i}^{2}\frac{\left\langle k^{2}\right\rangle }{{\left\langle k\right\rangle }^{2}}}\\ \;=\sqrt{k_{m}+\frac{{\left\langle k^{2}\right\rangle }^{2}}{{\left\langle k\right\rangle }^{2}}-\frac{k_{m}}{N}}. \end{array}$$ (14)

Note that the second moment $\left\langle k^{2}\right\rangle$ is a converging function of *m*. More specifically, we have

$$\langle k^{2}\rangle =r_{1}k_{1}^{2}\frac{1-{\left(ab^{2}\right)}^{-m}}{1-{\left(ab^{2}\right)}^{-1}},$$ (15)

with ${\left(ab^{2}\right)}^{-m}\to 0$ as m→∞.

With the derivation of Eq. (14), we now proceed to study the dependence of the ensemble average of the extreme eigenvalues on the mode number *m* of the multimodal networks. Here, we set $k_{1}=\langle k\rangle /2$ and $k_{m}=\sqrt{\langle k\rangle N}$. Figure 2 shows the ensemble average of the extreme eigenvalues for multimodal networks with *m* modes. It is observed that $\left\langle \lambda_{H}\right\rangle$ is the largest for bimodal network. It decreases gradually as *m* increases and eventually converges to a finite value. In consequence, the epidemic threshold for bimodal network is lower than that of the scale-free network, since the epidemic threshold is inversely proportional to the extreme eigenvalue. Thus, although it had been shown in Ref. 19 that bimodal network is optimal in terms of its tolerance against both random and targeted removal of nodes, epidemic spreading in this network is found to be less controllable.

To verify the accuracy of Eq. (14), we have compared its predicted values to those obtained numerically. For this, we have generated a scale-free network with $k_{1}=\langle k\rangle /2$ and $k_{m}=\sqrt{\langle k\rangle N}$ using the Barabási-Albert (BA) model.^2^ An ensemble with randomized network topology is then created using the degree-preserving algorithm of Ref. 28. For uncorrelated networks, we choose networks with assortativity coefficients near to zero. The maximum eigenvalue of each network is then computed and an ensemble average is obtained. In Fig. 3, we show the dependence of $\left\langle \lambda_{H}\right\rangle$ on ⟨k⟩ and *N*. Note that the numerical results are shown as squares. Next, we compute the ensemble average for multimodal networks with the same parameters using Eq. (14) by having $r_{m}\geq 1/N$, so that there is at least one node with degree $k_{m}$. In addition, we compare our results with those predicted from approximations provided by previous studies on $\left\langle \lambda_{H}\right\rangle$, i.e., $\langle \lambda_{H}\rangle =\sqrt{k_{H}}$^20,29–31^ and $\langle \lambda_{H}\rangle =\sqrt{k_{H}+\langle k^{2}\rangle /\langle k\rangle -1}$.^14^ As shown in Fig. 3, our results give values of $\left\langle \lambda_{H}\right\rangle$ that are much closer to the numerical results in comparison to those obtained based on the earlier approaches.

We have derived a more precise analytical approximation for the ensemble average of extreme eigenvalues for uncorrelated networks. However, many real-world networks are not uncorrelated, instead they show either assortative or disassortative mixing on their degree. For instance, the physics coauthorship network in Ref. 32 is assortative, while the world-wide web network is disassortative.^2^ For ensembles of network with identical degree distribution, $\left\langle \lambda_{H}\right\rangle$ of assortative networks which tend to link high-degree nodes to other high-degree nodes, are larger than $\left\langle \lambda_{H}\right\rangle$ of disassortative networks. For these networks, $k_{i}^{\left(1\right)}\propto k_{i}^{-\nu }$, with ν>0 for disassortative networks and ν<0 for assortative networks. Hence, although Eq. (14) gives ensemble average of $\lambda_{H}$ for randomly connected networks, it can be generalized to

$$\langle \lambda_{H}\rangle =\sqrt{k_{m}+\sum_{i=1}^{m}\frac{R_{i}k_{i}^{2-\nu }}{\left\langle k\right\rangle }}$$ (16)

for correlated networks. In fact, deviation in the extreme eigenvalue is larger in network ensemble with varying assortativities. Nonetheless, as shown in Ref. 14, fluctuation in the normalized extreme eigenvalue diminishes as the network size increases. In Fig. 4, we show the distribution of normalized extreme eigenvalue $\lambda_{H}^{N}$ for *N* = 1000, 3000, and 4000. Note that our results are obtained by first generating a BA network, before producing an ensemble through implementing ${\left(\sum_{i}k_{i}\right)}^{2}$ number of link rewirings which follow the constraints outlined in Ref. 28. As discussed in Ref. 14, degree correlations in the networks are generated through these constraints. As the network size grows, the distribution of $\lambda_{H}^{N}$ becomes more peaked and the standard deviation $\sigma^{N}$ decreases.

In conclusion, the ensemble averages of the extreme eigenvalues of scale-free networks can be determined more precisely through the multimodal networks with a large number of modes. Previous approximations on the extreme eigenvalue of adjacency matrix of random, undirected scale-free network have been analytically approximated up to the second order correction as $\lambda_{H}^{2}\approx k_{H}+k_{H}^{\left(1\right)}-1$,^14^ which is found to give better precision over results obtained through $\lambda_{H}^{2}\approx k_{H}$.^20,29–31^ Nonetheless, our results have clearly shown that the ensemble average of the extreme eigenvalues predicted by the second order correction is much too low, which can lead to an over-estimation of the epidemic threshold. When dealing with network dynamics such as the epidemic spreading of the community-acquired meticilin-resistant *Staphylococcus aureus* (CA-MRSA) superbugs that are resistant to many antibiotics,^33^ such an over-estimation of the epidemic threshold can lead to serious consequences. In view of this, the analytical solution derived from the multimodal network which is able to provide closer approximation to the ensemble average of extreme eigenvalue of scale-free network is important. We have demonstrated that our analytical approximation predicted accurately the ensemble average of the extreme eigenvalues for scale-free networks with β≈3 and $k_{m}=\sqrt{N\langle k\rangle }$. In fact, Eq. (14) is valid for a broad class of scale-free networks with different values of β and $k_{m}$. While $k_{m}$ is a free parameter, the exponent β can be adjusted by tuning the parameters *a* and *b* through the relation: β=-lna/lnb+1.^19^ From Eq. (14), it is clear that $\left\langle \lambda_{H}\right\rangle$ increases with an increase in $\sqrt{k_{m}}$. Furthermore, it can be deduced from Eq. (14) that $\left\langle \lambda_{H}\right\rangle$ reduces as β increases. This results from a decrease in the variance of the degree and the fraction of high degree nodes in the network, as the exponent β increases.

## Figures and Tables

**FIG. 1.**
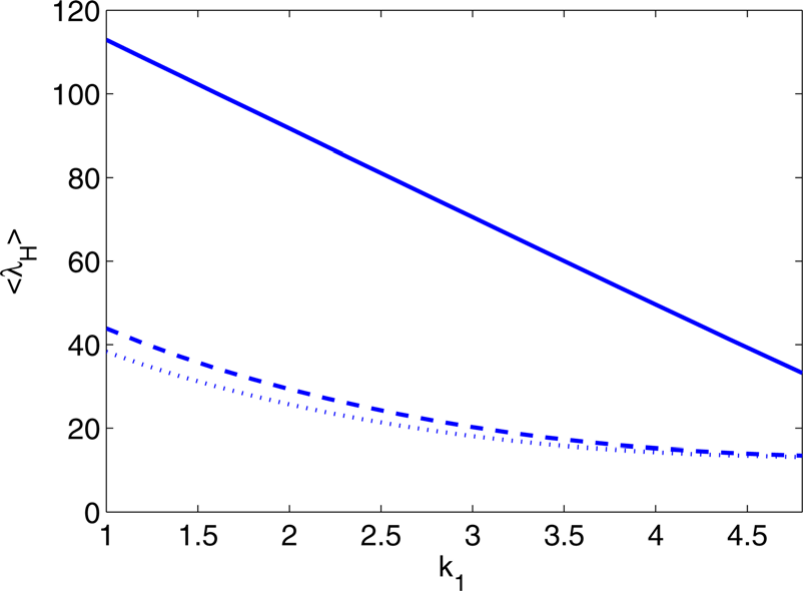
Dependence of $\left\langle \lambda_{H}\right\rangle$ on $k_{1}$ for multimodal network with $\langle k\rangle =6,N=3\times 10^{3}$ and *m* = 2 (solid line), 10 (dashed line), and 21 (dotted line).

**FIG. 2.**
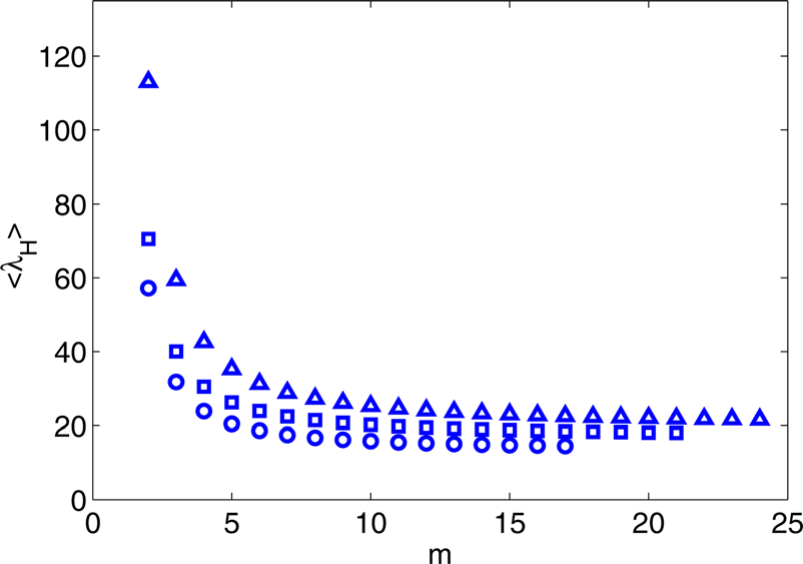
Dependence of $\left\langle \lambda_{H}\right\rangle$ on *m* for multimodal network with $k_{1}=\langle k\rangle /2$. Note that the average degree and size of the network are: (1) ⟨k⟩=4,N=3000 (circles), (2) ⟨k⟩=6,N=3000 (squares), and (3) ⟨k⟩=6,N=8000 (triangles).

**FIG. 3.**
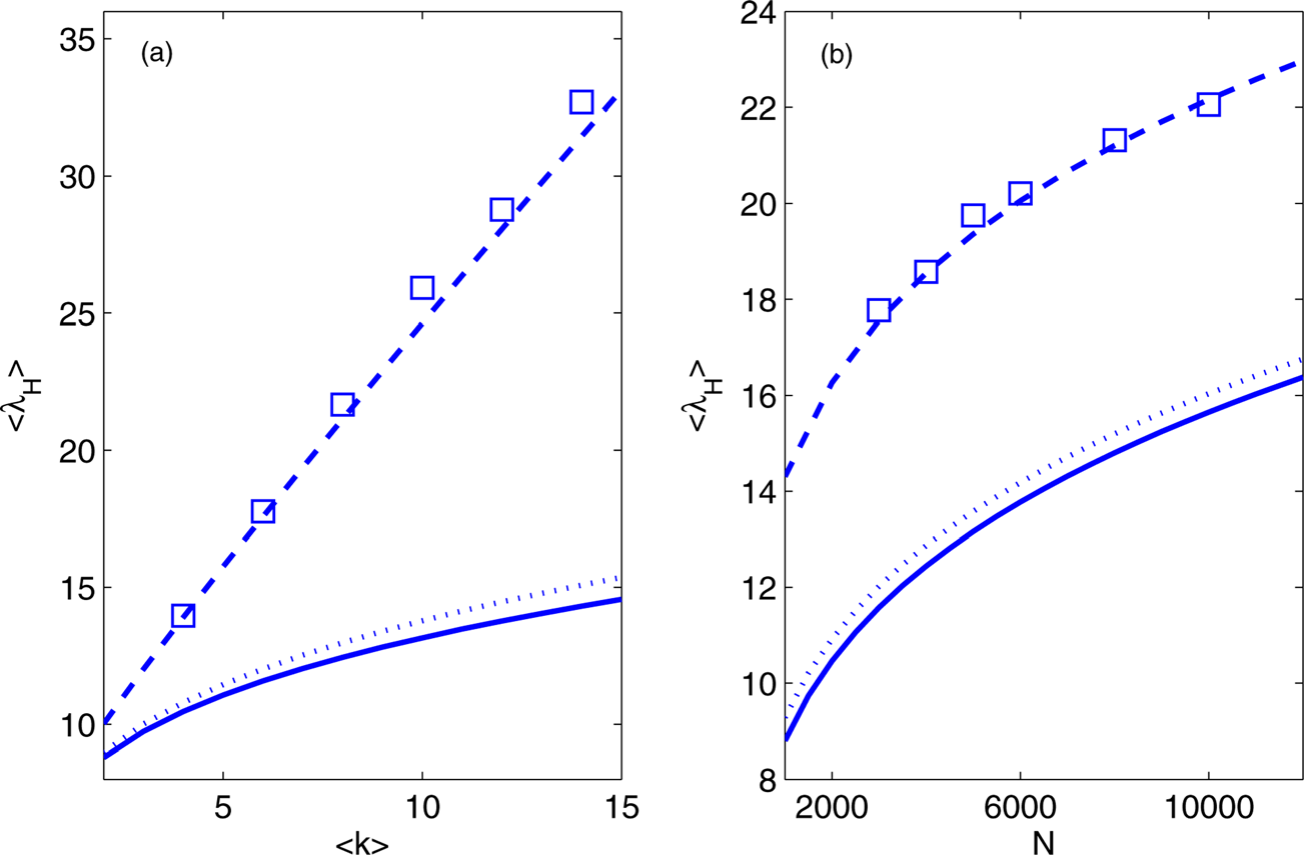
Dependence of $\left\langle \lambda_{H}\right\rangle$ on (a) ⟨k⟩ and (b) *N*, for an ensemble of scale-free networks with $k_{1}=\langle k\rangle /2$. Note that $N=3\times 10^{3}$ for (a) and ⟨k⟩=6 for (b). The results for $\langle \lambda_{H}\rangle =\sqrt{k_{H}}$ and $\langle \lambda_{H}\rangle =\sqrt{k_{H}+\langle k^{2}\rangle /\langle k\rangle -1}$ are shown as solid curves and dotted curves, respectively, while the analytical results from Eq. (14) are shown as dashed curves. The numerical results from the BA model, which are obtained after averaging over 200 network realizations, are shown as squares.

**FIG. 4.**
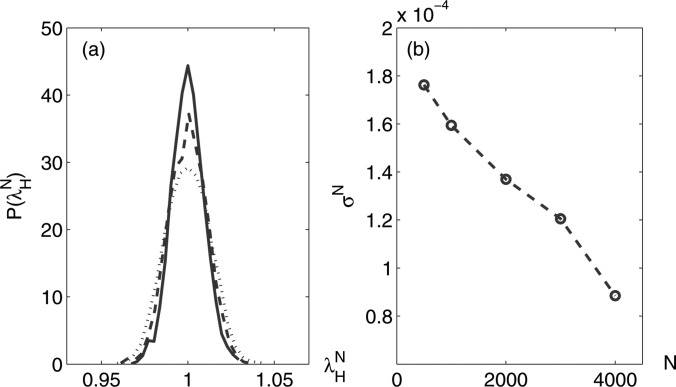
Numerical results for (a) the distribution $P(\lambda_{H}^{N})$ of the normalized extreme eigenvalue $\lambda_{H}^{N}$ for *N* = 1000 (dotted line), 3000 (dashed line), 4000 (solid line), and (b) the *N* dependence of the corresponding standard deviation $\sigma^{N}$. Note that all the network ensembles consist of realizations of 5000 networks with ⟨k⟩=6 and $k_{1}=3$.
